# Ectopic fat is associated with cardiac remodeling—A comprehensive assessment of regional fat depots in type 2 diabetes using multi-parametric MRI

**DOI:** 10.3389/fcvm.2022.813427

**Published:** 2022-07-28

**Authors:** Carl Edin, Mattias Ekstedt, Tobias Scheffel, Markus Karlsson, Eva Swahn, Carl Johan Östgren, Jan Engvall, Tino Ebbers, Olof Dahlqvist Leinhard, Peter Lundberg, Carl-Johan Carlhäll

**Affiliations:** ^1^Division of Diagnostics and Specialist Medicine, Department of Health, Medicine and Caring Sciences, Linköping University, Linköping, Sweden; ^2^Department of Gastroenterology in Linköping and Department of Biomedical and Clinical Sciences, Linköping University, Linköping, Sweden; ^3^Center for Medical Image Science and Visualization, Linköping University, Linköping, Sweden; ^4^AMRA Medical AB, Linköping University, Linköping, Sweden; ^5^Department of Radiation Physics and Department of Health, Medicine and Caring Sciences, Linköping University, Linköping, Sweden; ^6^Department of Cardiology in Linköping and Department of Health, Medicine and Caring Sciences, Linköping University, Linköping, Sweden; ^7^Division of Prevention, Rehabilitation and Community Medicine, Department of Health, Medicine and Caring Sciences, Linköping University, Linköping, Sweden; ^8^Department of Clinical Physiology in Linköping and Department of Health, Medicine and Caring Sciences, Linköping University, Linköping, Sweden

**Keywords:** ectopic fat, left ventricular structure, left ventricular diastolic function, cardiac remodeling, magnetic resonance imaging, type 2 diabetes, visceral fat

## Abstract

**Background:**

Different regional depots of fat have distinct metabolic properties and may relate differently to adverse cardiac remodeling. We sought to quantify regional depots of body fat and to investigate their relationship to cardiac structure and function in Type 2 Diabetes (T2D) and controls.

**Methods:**

From the SCAPIS cohort in Linköping, Sweden, we recruited 92 subjects (35% female, mean age 59.5 ± 4.6 years): 46 with T2D and 46 matched controls. In addition to the core SCAPIS data collection, participants underwent a comprehensive magnetic resonance imaging examination at 1.5 T for assessment of left ventricular (LV) structure and function (end-diastolic volume, mass, concentricity, ejection fraction), as well as regional body composition (liver proton density fat fraction, visceral adipose tissue, abdominal subcutaneous adipose tissue, thigh muscle fat infiltration, fat tissue-free thigh muscle volume and epicardial adipose tissue).

**Results:**

Compared to the control group, the T2D group had increased: visceral adipose tissue volume index (*P* < 0.001), liver fat percentage (*P* < 0.001), thigh muscle fat infiltration percentage (*P* = 0.02), LV concentricity (*P* < 0.001) and LV E/e'-ratio (*P* < 0.001). In a multiple linear regression analysis, a negative association between liver fat percentage and LV mass (St Beta −0.23, *P* < 0.05) as well as LV end-diastolic volume (St Beta −0.27, *P* < 0.05) was found. Epicardial adipose tissue volume and abdominal subcutaneous adipose tissue volume index were the only parameters of fat associated with LV diastolic dysfunction (E/e'-ratio) (St Beta 0.24, *P* < 0.05; St Beta 0.34, *P* < 0.01, respectively). In a multivariate logistic regression analysis, only visceral adipose tissue volume index was significantly associated with T2D, with an odds ratio for T2D of 3.01 (95% CI 1.28–7.05, *P* < 0.05) per L/m^2^ increase in visceral adipose tissue volume.

**Conclusions:**

Ectopic fat is predominantly associated with cardiac remodeling, independently of type 2 diabetes. Intriguingly, liver fat appears to be related to LV structure independently of VAT, while epicardial fat is linked to impaired LV diastolic function. Visceral fat is associated with T2D independently of liver fat and abdominal subcutaneous adipose tissue.

## Introduction

Visceral adipose tissue (VAT) is associated with metabolic cardiovascular disease risk factors (1, 2), above and beyond the abdominal subcutaneous adipose tissue (ASAT). Data from cardiovascular imaging studies also show that the volume of VAT is associated with subclinical cardiac remodeling, in terms of increased left ventricular (LV) concentricity and impaired diastolic relaxation (3–5). Fat can also accumulate in and around normally lean tissues, such as the heart, skeletal muscle and liver, a process termed 'ectopic fat infiltration' (6). In addition to VAT, both hepatic and cardiac ectopic fat depots have previously been linked to increased cardiovascular risk (7, 8), as well as adverse LV structural remodeling and dysfunction (9–11).

How different fat depots affect cardiovascular risk factors is still unclear. Ectopic and visceral fat depots are thought to act through both local and systemic effects (6), and probably affect the cardiovascular system differently. Based on data from the Framingham Heart Study, Lee et al. demonstrated that VAT and liver fat were more strongly associated with cardiometabolic risk factors, compared to other sites for ectopic fat accumulation (12). In contrast, a recent analysis from the Multi-Ethnic Study of Atherosclerosis found that the total amount of pericardial fat, but not hepatic fat, was independently associated with risk of atherosclerotic events over a median of 12.2 years follow-up (13).

Since VAT is related to the overall burden of ectopic fat (14), it might not constitute the primary mediator for adverse cardiac remodeling. Given the close interrelationship between VAT, ectopic fat and gluco-metabolic disease, the causal pathways need to be explored in more detail. Thus, there is a need for studies performing more comprehensive analyses of regional fat depots. Magnetic resonance imaging (MRI) allows for accurate assessment of whole-body adipose tissue, including accurate assessment of liver fat, which can be challenging with other imaging modalities (15). Further studies also need to consider T2D in order to distinguish the characteristics of separate fat depots, as diabetes *per se* is likely to affect cardiac remodeling (16).

In the present study, advanced MRI techniques were utilized to accurately assess whole-body adipose tissue, including liver fat, epicardial fat, visceral fat, abdominal subcutaneous fat, and skeletal muscle fat in participants with T2D and matched controls. We sought to investigate the relationship between these regional depots of fat to measures of LV structure and function as well as to T2D. We hypothesized that regional fat depots differ in their associations to cardiac remodeling and T2D.

## Materials and methods

### Study design and population

We conducted an exploratory cross-sectional study that was a sub-study within SCAPIS (17). We included 92 participants in the ages 50–64 years from the SCAPIS cohort in Linköping. We first included 46 participants with T2D. They were identified as having T2D according to health forms, fP-glucose ≥7.0 mmol/L on two occasions or HbA1c ≥ 48 mmol/mol. Additionally, 46 control subjects were recruited, reported as not having T2D according to the health forms, fP-glucose <6.0 mmol/L and HbA1c <42 mmol/mol. The control subjects were matched to the diabetes group on an individual basis with regard to age, sex, and smoking. General exclusion criteria were contraindications to MRI and significantly irregular ventricular rhythm.

### Ethics

All participants provided written informed consent to participate. The study was performed in accordance with the Declaration of Helsinki and approved by the Linköping Ethical Review Board (Dnr 2016/229-31).

### SCAPIS data

From the core SCAPIS data in Linköping, we had access to the following data:

- Demographic variables (sex, age, alcohol consumption, and smoking)
- Self-reported cardiovascular morbidity and drug use
- Anthropometric measurements (body weight, height, and BMI)
- Routine clinical biochemistry (glucose status, lipid status, highly sensitive CRP)
- Echocardiography (E/A-ratio, E/e'-ratio).

Echocardiography was performed using a GE Vivid E95 rev 201 or E9 (GE Vingmed Ultrasound, Horten, Norway) according to current guidelines from EACVI/ASE (18) (see Supplementary material for details on data acquisition and analysis).

### Acquisition of MRI data

**Figure 1:**
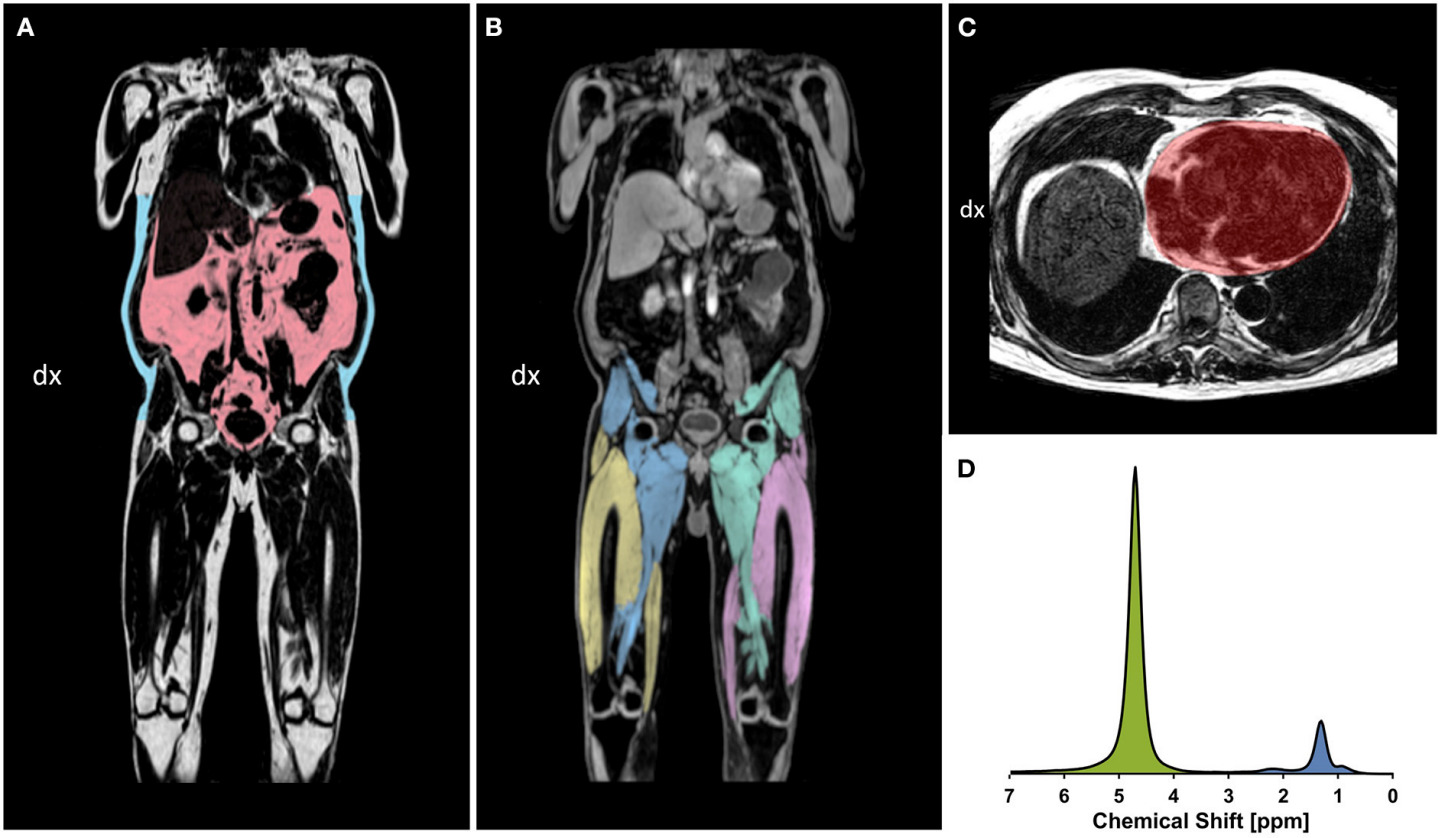
**(A,B)** example coronal slices of whole-body Dixon images. **(A)** Fat images with the VAT (red) and ASAT segmented (blue). **(B)** Water images with the segmented thigh muscle groups: right posterior thigh (blue), right anterior thigh (yellow), left posterior thigh (green), and left anterior thigh (pink). **(C)** Cardiac fat image in transversal view from the 3D Dixon sequence, showing the segmented region of interest (red) following the epicardial border. **(D)** Liver H-1 MR spectrum obtained at 1.5 T, showing the water resonance at 4.76 ppm (green), and the major fatty-acyl chain resonances at 1.21 ppm (methylene), as well as 0.9 ppm (methyl) and 2.2 ppm (alpha-olefinic etc) (blue). All lipid resonances were included in the integration procedure.

After inclusion, all 92 participants underwent a comprehensive MRI examination at Linköping University Hospital between November 2017 and July 2018. Data were acquired in a single examination using a Philips Achieva dStream 1.5 T MRI-scanner (Philips Healthcare, Best, The Netherlands) and included a neck-to-knee four-echo mDixon sequence, liver proton magnetic resonance spectroscopy (^1^H-MRS) sequence, cardiac 3D cine balanced steady state-free precession (bSSFP) sequence and cardiac 3D Dixon sequence. Figure 1 shows example images from the whole-body and cardiac Dixon sequences as well as the liver H-1 spectroscopy sequence. See Supplementary material for details on the magnetic resonance sequences.

### Analysis of MRI data

#### Abdominal and thigh composition

Images from the neck-to-knee mDixon sequence were analyzed and automatically segmented using the AMRA^®^ Researcher (AMRA Medical AB, Linköping, Sweden) post-processing software. Briefly, the image analysis consisted of (1) image calibration, (2) fusion of image stacks, (3) image segmentation, and (4) quantification of fat and muscle volumes (15, 19, 20) and included manual quality control by a blinded trained operator. The following parameters were obtained:

- Abdominal subcutaneous adipose tissue (ASAT): the subcutaneous adipose tissue volume in the abdomen from the top of the femoral head to the top of the thoracic vertebra T9.
- Visceral adipose tissue (VAT): the adipose tissue volume within the abdominal cavity, excluding adipose tissue outside the abdominal skeletal muscles and adipose tissue and lipids within the cavity and posterior of the spine and back muscles.
- Fat-tissue free muscle volume (FFMV): the volume of all voxels with a fat fraction below 50%, i.e., “viable muscle tissue”, in the thighs.
- Muscle fat infiltration (MFI): the mean fat fraction in the FFMV of the right and left anterior thigh.

VAT, ASAT and FFMV were indexed to height squared (21) and denoted with the suffix “-i”, e.g. VATi.

#### Liver fat infiltration

To quantify liver fat infiltration, we measured 'Proton Density Fat Fraction' (PDFF) using MRS, which provides good precision on low fat concentrations and robustness toward artifacts. PDFF was measured using a ^1^H-MRS PRESS-sequence as described in previous publications (22, 23). Post-processing of the MRS data was performed by quantifying the integrals of water and fat resonances, respectively, using jMRUI and the AMARES algorithm (24, 25). In analyzing the spectra, we included all resonances representing different lipid moieties. The integrals were also corrected for systematic differences in T_1_ and T_2_ for fat and water, which were assumed to be as follows T_1_: 236 ms (fat) and 663 ms (water) and T_2_: 58.5 ms (fat) and 43.4 ms (water) (22, 26, 27).

#### Epicardial adipose tissue

The epicardial adipose tissue (EAT) volumes were determined by analyzing the transversal image stacks from the cardiac 3D Dixon sequence. ROI-drawing was performed using the open-source software MITK (version 2018.04.2) (28). Segmentation was performed in end-diastole from the most caudal slice where myocardium was visible to the first slice in the cranial direction where the lumen of right branch of the pulmonary artery displayed continuity. The adipose tissue volumes were then calculated using a separate script in MATLAB (MathWorks, Natick, MA), as described by Homsi et al. (29).

#### Left ventricular structure and function

Images from the cardiac 3D cine bSSFP sequence were analyzed using software Segment CMR (version 3.2 R8452, Medviso AB, Lund, Sweden) (30), to acquire structural (mass, end-diastolic volume and concentricity) and functional (stroke volume and ejection fraction) LV measures. To do this, epicardial and endocardial borders of the LV myocardium were segmented manually at end-diastole and end-systole using short axis images. The papillary muscles were regarded as part of the blood pool and LV mass (LVM) was acquired from the end-diastolic segmentation.

Stroke volume (SV) was calculated as: stroke volume = end-diastolic volume – end-systolic volume. Ejection fraction (EF) was calculated as: ejection fraction = stroke volume / end-diastolic volume. As a measure of concentricity, the ratio between LV mass and LV end-diastolic volume (LVEDV) was calculated for each participant, termed LV concentricity (LVC). Where applicable, cardiac parameters (including epicardial adipose tissue) were indexed to body surface area using the Mosteller method (31): BSA (m^2^) = (height (cm) × weight (kg)/3,600)^1/2^. Just as body composition variables, indexed cardiac parameters were denoted with the suffix “-i”.

### Statistics

For all statistical analyses, SPSS IBM version 26 was used. A *P*-value of <0.05 was considered statistically significant. Normality of distribution was assessed using the Kolmogorov-Smirnoff test. For group comparisons of continuous variables, independent sample *T*-test was used for parametric and Mann-Whitney U test was used for non-parametric variables. Chi-square tests were used to compare categorical variables. A Bonferroni correction was used for group comparison of the MRI-derived body composition parameters as well as the structural and functional LV parameters.

To test the associations between cardiac remodeling and fat infiltration, linear regression analyses were performed. Structural and functional LV variables were deployed as dependent variables and the metrics of fat as independent variables. Regression analysis was performed in three steps. The crude regression model (*Crude model*) was a simple linear regression between each fat parameter and each LV parameter. *Model 1* was a multiple linear regression with each fat parameter deployed separately, adjusted for sex and diabetes. *Model 2* was a multiple linear regression with liver fat, epicardial fat and visceral fat deployed simultaneously in a single model, adjusted for sex and diabetes. MFI data were missing in 25% of the participants and was therefore not used in *Model 2*. Liver fat distribution was skewed despite stratifying for sex and diabetes, and therefore logarithmically transformed for this analysis. For every model we calculated the overall *R*^2^-value as well as *P*-value and standardized beta coefficients for each independent variable.

A logistic regression analysis was performed with the measures of body composition as predictors and the state of T2D as outcome. In the univariate model, each variable was deployed separately. We also performed a multivariate model including liver fat, visceral and abdominal subcutaneous fat simultaneously.

## Results

### Group comparison of demographic and basic clinical data

Between the groups, there were no statistical differences in age, sex, smoking, prevalence of frequent alcohol consumption or self-reported cardiac disease. The T2D group displayed significantly higher BMI (29.6 vs. 26.2 kg/m^2^, *P* < 0.001), higher P-glucose (8.4 vs. 5.3 mmol/L, *P* < 0.001), higher HbA1c (54 vs. 34 mmol/mol, *P* < 0.001) compared to the normoglycemic group. The T2D group also displayed significantly lower total cholesterol (4.3 vs. 5.7 mmol/L, *P* < 0.001), lower LDL cholesterol (2.2 vs. 3.6 mmol/L, *P* < 0.001) and higher self-reported usage of lipid-lowering medication (41.3 vs. 6.5 %, *P* < 0.001) (see Table 1).

**Table 1:** Group comparison of demographic and basic clinical data.

	**T2D**	**Controls**	***P*** **-value**
	**(*****n** **=*** **46)**	**(*****n** **=*** **46)**	
**Age (years)**	**59.6 ± 4.6**	**59.3 ± 4.7**	**0.77**
Sex (women)	16 (34.8%)	16 (34.8%)	–
Smoking	4 (8.7%)	4 (8.7%)	–
Frequent alcohol consumption*	3 (6.5%)	5 (10.9%)	0.46
Self-reported cardiac disease**	8 (17.4%)	3 (6.5%)	0.11
Time since diabetes diagnosis (years)	6.4 ± 5.5	–	–
Insulin use	10 (21.7%)	–	–
Anti-hypertensive medication	23 (50%)	5 (10.9 %)	<0.001
Lipid-lowering medication	19 (41.3%)	3 (6.5%)	<0.001
Weight (kg)	88.9 ± 13.7	80.7 ± 12.3	<0.01
Height (m)	1.73 ± 0.09	1.75 ± 0.09	0.21
BMI (kg/m^2^)	29.6 ± 3.3	26.2 ± 3.2	<0.001
Heart rate (BPM)	73 ± 13	66 ± 10	0.02
Systolic blood pressure (mm Hg)	144 ± 15	137 ± 17	0.07
Diastolic blood pressure (mm Hg)	86 ± 13	82 ± 10	0.07
Capillary P-glucose (mmol/L)	8.4 ± 2.3	5.3 ± 0.5	<0.001
HbA1c (mmol/mol)	54 ± 16	34 ± 3	<0.001
Total cholesterol (mmol/L)	4.3 ± 1.1	5.7 ± 1.2	<0.001
HDL cholesterol (mmol/L)	1.4 ± 0.5	1.6 ± 0.5	0.01
LDL cholestrol (mmol/L)	2.2 ± 1.0	3.6 ± 1.0	<0.001
Triglycerides (mmol/L)	1.7 ± 1.1	1.2 ± 0.6	0.02
hsCRP (mg/L)	2.0 ± 2.2	2.1 ± 2.8	0.99

*Data shown as mean ± SD or n (%). T2D, type 2 diabetes; BP, blood pressure; HDL, high density lipoprotein; LDL, low density lipoprotein; hsCRP, high-sensitivity C-reactive protein*.

*Regular consumption of more than three times weekly.

**History of coronary heart disease, atrial fibrillation, heart failure or valvular heart disease.

### Group comparison of body composition parameters

As compared to controls the T2D group had a significantly higher VATi volume (1.94 vs. 1.35 L/m^2^, *P* < 0.001), higher liver fat percentage (11.7 vs. 5.7 %, *P* < 0.001) and MFI percentage (9.4 vs. 7.7 %, *P* = 0.02) (see Table 2).

**Table 2:** Group comparison of regional body composition parameters.

	**T2D (*****n** =* **46)**	**Controls (*****n** =* **46)**	***P*** **-value**
EATi (mL/m^2^)	62.1 ± 21.0	51.6 ± 17.7	0.07
Liver fat (%)	11.7 ± 9.0	5.7 ± 6.8	<0.001
VATi (L/m^2^)	1.94 ± 0.74	1.35 ± 0.63	<0.001
ASATi (L/m^2^)	2.73 ± 1.03	2.33 ± 1.09	0.20
FFMVi (L/m^2^)	3.86 ± 0.67	3.78 ± 0.59	1.00
MFI (%)	9.4 ± 2.5	7.7 ± 2.3	0.02

*Data shown as mean ± SD. EATi, epicardial adipose tissue index; VATi, visceral adipose tissue index; ASATi, abdominal adipose tissue index; FFMVi, fat tissue-free muscle volume index; MFI, muscle fat infiltration*.

### Group comparison of LV parameters

The T2D group displayed a higher E/e'-ratio (13.4 vs. 10.1, *P* < 0.001), indicating impaired diastolic function, and increased LV concentricity, (0.91 vs. 0.79 g/mL, *P* < 0.001). There were no significant differences in ejection fraction, LVEDVi or LVMi (see Table 3).

**Table 3:** Group comparison of LV structure and function.

	**T2D (*****n** =* **46)**	**Controls (*****n** =* **46)**	**P-value**
LVMi (g/m^2^)	62.2 ± 12.5	59.0 ± 9.74	1.00
LVEDVi (mL/m^2^)	69.6 ± 15.2	76.0 ± 15.6	0.33
LVC (g/mL)	0.91 ± 0.12	0.79 ± 0.12	<0.001
LVSVi (mL/m^2^)	40.9 ± 9.1	44.2 ± 8.3	0.54
LV ejection fraction (%)	59.9 ± 9.2	58.6 ± 6.9	1.00
LV E/A-ratio	1.06 ± 0.49	1.09 ± 0.33	1.00
LV E/e'-ratio	13.4 ± 4.7	10.1 ± 2.3	<0.001

*Data shown as mean ± SD. LVMi, left ventricular mass index; LVEDVi, left ventricular end-diastolic volume index; LVC, left ventricular concentricity; LVSVi, left ventricular stroke volume index; E, early diastolic velocity of mitral inflow; A, late diastolic velocity of mitral inflow; e', early diastolic mitral annular velocity*.

### Linear regression analyses

**Figure 2:**
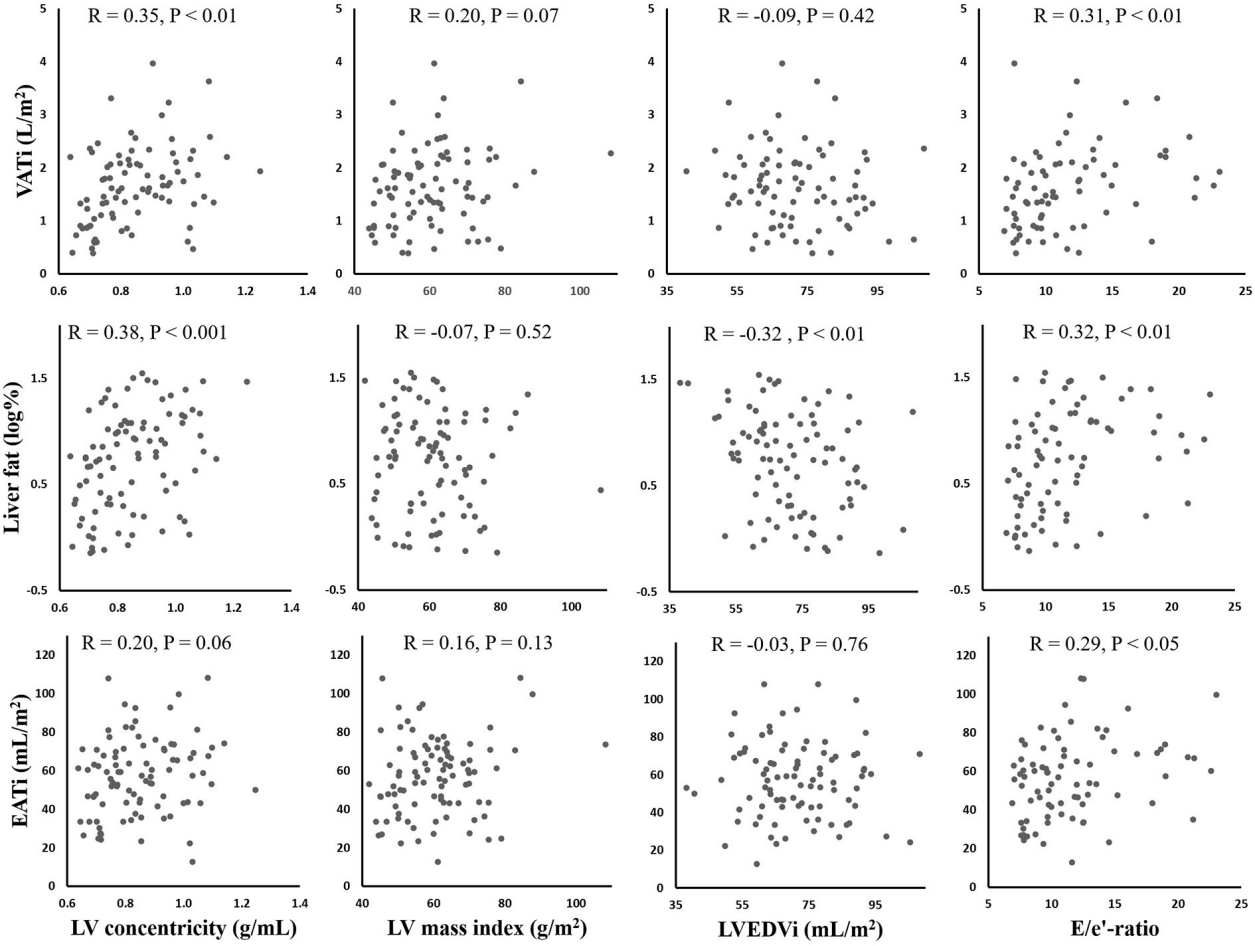
Scatterplots showing the correlations between fat depots (Y-axes) and left ventricular parameters (X-axes). Pearson's correlation coefficients (R) and P-values are provided for each plot. VATi, visceral adipose tissue index; EATi, epicardial adipose tissue index; LV, left ventricular; EDVi, end-diastolic volume index; E, early diastolic velocity of mitral inflow; e', early diastolic mitral annular velocity.

In general, liver fat was associated with structural LV parameters. In the *Crude model* (not shown in tables), there was a positive relationship between liver fat and LVC (standardized beta=0.381, *P* < 0.001). The association was still significant when including diabetes and sex (standardized beta= 0.229, *P* < 0.05), but not when including VATi and EATi (*Model 2*). The negative associations of liver fat to LVMi and LVEDVi were still significant in *Model 2, P* < 0.05 for both. All parameters of fat had a significant positive relationship with E/e'-ratio in the *Crude model*. When adjusting for diabetes, only EATi and ASATi were significantly related to E/e', *P* < 0.05 for both. VATi was positively associated with both LVC and E/e' in the *Crude model*, although these associations were non-significant when adjusting for diabetes and sex (*Model 1*). Table 4 displays the results from the linear regression analyzes in model 1 and 2. Figure 2 shows scatterplots for the crude associations between liver fat, EATi and VATi, and LV parameters.

**Table 4:** Linear regression analyses using two different models.

	**LVC (g/mL)**			**LVMi (g/m** ^2^ **)**			**LVEDVi (mL/m** ^2^ **)**			**LV E/e'-ratio**			**LV ejection fraction (%)**		
	**St Beta**	*R* ^2^	***P*** **-value**	**St Beta**	*R* ^2^	***P*** **-value**	**St Beta**	*R* ^2^	***P*** **-value**	**St Beta**	*R* ^2^	***P*** **-value**	**St Beta**	*R* ^2^	***P*** **-value**
VATi (L/m^**2**^)		
*Model 1*	0.19	0.22	0.11	−0.14	0.43	0.17	−0.27	0.30	<0.05	0.23	0.24	0.06	0.15	0.07	0.24
*Model 2*	0.11	0.23	0.54	−0.03	0.46	0.85	−0.10	0.33	0.53	0.05	0.26	0.81	0.15	0.08	0.42
Liver fat (log%)		
*Model 1*	0.23	0.24	<0.05	−0.22	0.45	<0.05	−0.33	0.36	<0.01	0.19	0.20	0.11	0.13	0.08	0.25
*Model 2*	0.13	0.23	0.33	−0.23	0.46	<0.05	−0.27	0.33	<0.05	0.12	0.26	0.42	0.06	0.08	0.69
EATi (mL/m^**2**^)		
*Model 1*	0.07	0.20	0.51	−0.04	0.41	0.63	−0.12	0.29	0.24	0.24	0.22	<0.05	0.02	0.07	0.88
*Model 2*	-0.01	0.23	0.95	0.06	0.46	0.60	0.02	0.33	0.90	0.18	0.26	0.21	−0.06	0.08	0.68
MFI (%)		
*Model 1*	0.20	0.27	0.13	0.03	0.44	0.76	−0.14	0.37	0.25	0.22	0.20	0.12	0.05	0.08	0.72
ASATi (L/m^**2**^)		
*Model 1*	0.19	0.21	0.13	−0.18	0.43	0.10	−0.30	0.30	<0.05	0.34	0.29	<0.01	0.20	0.07	0.14

*Model 1: included each fat predictor separately plus sex and diabetes. Model 2: included liver fat, VATi and EATi (in the same model) plus sex and diabetes. St Beta, standardized beta coefficient; EATi, epicardial adipose tissue index; ASATi, abdominal subcutaneous adipose tissue index; VATi, visceral adipose tissue index; MFI, thigh muscle fat infiltration; LV, left ventricular; LVC, left ventricular concentricity; LVMi, left ventricular mass index; LVEDVi, left ventricular end-diastolic volume index; E, early diastolic velocity of mitral inflow; e', early diastolic mitral annular velocity*.

For *Model 1* and *Model 2*, results were consistent when replacing diabetes status with BMI (not shown in tables). When adding adjustment for self-reported cardiac disease to sex and diabetes (not shown in tables), results in *Model 1* and *2* were overall directionally consistent. Results in *Model 1* persisted. Associations in *Model 2* were slightly attenuated, as liver fat was borderline significant as predictor for LVMi (standardized beta = −0.20, *P* = 0.070) and LVEDVi (standardized beta = −0.25, *P* = 0.053).

### Logistic regression analysis

In the univariate analyses, EATi, VATi, liver fat and MFI were significantly associated to T2D (*P* < 0.05 for all). In the multivariate model (including ASATi, VATi and liver fat), only VATi remained statistically significant (*P* < 0.05), with an odds ratio (OR) increase for T2D of 3.01 for each L/m^2^ increment in VATi (see Table 5).

**Table 5:** Logistic regression analyses.

	**Univariate models**		**Multivariate model**	
	OR for diabetes (95% CI)	*P*-value	OR for diabetes (95% CI)	*P*-value
EATi (per mL/m^2^ increase)	1.03 (1.01–1.05)	<0.05	–
ASATi (per L/m^2^ increase)	1.45 (0.94–2.24)	0.09	1.28 (0.82–2.01)	0.28
VATi (per L/m^2^ increase)	3.73 (1.75–7.95)	<0.001	3.01 (1.28–7.05)	<0.05
Liver fat (per % point increase)	1.11 (1.04–1.18)	<0.01	1.03 (0.96–1.11)	0.46
MFI (per % point increase)	1.38 (1.09–1.75)	<0.01	–
FFMVi (per L/m^2^ increase)	1.26 (0.63–2.50)	0.52	–

*Univariate models show each predictor deployed separately. The multivariate models display ASATi, VATi and liver fat deployed simultaneously. OR, odds ratio; EATi, epicardial adipose tissue index; ASATi, abdominal subcutaneous adipose tissue index; VATi, visceral adipose tissue index; MFI, thigh muscle fat infiltration; FFMVi, fat-tissue free muscle volume index*.

## Discussion

In our well-characterized study population, we found that liver fat infiltration, but not visceral fat, was related to structural LV parameters, including negative relationships to chamber mass and end-diastolic volume. Among the visceral and ectopic fat depots analyzed, the epicardial fat volume was the only parameter associated with diastolic function when adjusting for diabetes and sex. Visceral fat was significantly related to T2D, independently of liver fat and abdominal subcutaneous fat.

### Association between regional fat and LV structure and function

Several large population-based studies have previously reported significant associations between visceral adipose tissue (VAT) and structural LV parameters. Neeland et al. (4) demonstrated that VAT was linked to increased LV concentricity and lower LVEDV. Other studies have reported positive relationships between VAT and LV concentricity (5), and negative relationships between VAT and LVEDV (32). Noticeably, neither of these studies analyzed or accounted for liver fat infiltration. A study that did analyze both VAT and liver fat was published by Schlett et al. (3). The authors found that both measures of fat were positively associated with LV concentricity and negatively with LVEDV, independently of diabetes. However, Schlett et al. did not analyze VAT and liver fat in the same regression model, which is a possible explanation for the discrepancy to our results. Our findings could indicate that liver fat acts as an interlink for the previously reported associations between VAT and LV structural remodeling, possibly due to the potentially progressive and inflammatory nature of fatty liver disease. For example, it has been demonstrated that patients with non-alcoholic fatty liver disease, especially non-alcoholic steatohepatitis, have increased systemic levels of inflammatory cytokines (33). Individuals with hepatic steatosis have also been shown to have altered LV metabolism in terms of reduced phosphocreatinine/adenosine triphosphate ratio, while still having preserved LV function (34).

Interestingly, while liver fat was related to LV structural parameters in our study, it was not significantly associated with any functional LV measures after adjustment for sex and diabetes. Among the visceral and ectopic fat depots, EAT was the only parameter in model 1 that was significantly positively associated with E/e' ratio, an indicator of LV diastolic dysfunction. Although this association was non-significant in model 2, perhaps due to lack of power, our results support previous findings that epicardial fat relates to LV function without a significant association to structural remodeling. A meta-analysis stated that EAT was associated with LV diastolic dysfunction, but the associations between EAT and LV structure were found to be largely inconsistent and uncertain (10). There are several postulated ways through which EAT could impair cardiac ventricular diastolic function. For example, through paracrine inflammatory mechanisms, molecular lipotoxic effects of free fatty acids (35, 36) as well as by physical obstructive effects (37). Given the close relationship between cardiac fat depots, epicardial fat may also act as a marker of intramyocardial triglyceride content, which too has been linked to impairment of LV function (36, 38–40). Finally, it is possible that altered hemodynamic factors, such as heart rate and blood pressure, influence the associations between ectopic fat infiltration and cardiac remodeling.

Diastolic impairment was the main cardiac functional abnormality found in this study population, related to both diabetes and to increasing volumes of EAT (independently of diabetes), while there were no significant findings regarding ejection fraction between the groups or in the regression analyses. Moreover, lower LVEDV, lower LVM and higher LV concentricity in the left ventricle was associated with liver fat. Taken together, these findings closely resemble the cardiac phenotype seen in heart failure with preserved ejection fraction, where patients are typically seen with preserved or increased ejection fraction and low or normal LVEDV (41). The role of ectopic fat in the development of heart failure with preserved ejection fraction may warrant further research, especially since previous studies have recognized obesity as a risk factor for this type of heart failure (42).

### Association between regional fat and metabolic disease

The high proportion of individuals with T2D and matched design makes our study well suited to assess the relationship between individual regional fat depots and T2D using logistic regression. In the univariate models we found VAT as well as EAT, liver fat and MFI, but not ASAT, to be significant predictors of T2D. This is in line with the generally held view that ASAT and VAT have distinct metabolic and endocrine properties (43). And again, since visceral fat is closely related to the overall burden of ectopic fat (14), it is not surprising that both VAT and liver fat were significant predictors of T2D in our univariate analysis. In the multivariate analysis (including ASAT, VAT, and liver fat) VAT was a significant predictor of T2D while liver fat was non-significant. It is interesting that VAT, but not liver fat, was a significant predictor since several previous studies have demonstrated an independent role of fatty liver disease in T2D development (44, 45). It should be emphasized, however, that our analysis was employed to assess associations to clinically manifest disease. More sensitive and non-dichotomous measures of gluco-metabolic derangement, such as insulin sensitivity index, could yield additional insight into the roles of hepatic and visceral fat in development of metabolic disease. Nonetheless, our findings do support the hypothesis that different regional fat depots have dissimilar effects on cardiovascular and metabolic health.

### Limitations

Due to the relatively small sample size, our data may lack statistical power to detect additional significant relationships between measures of fat, cardiac parameters and T2D. The rather small sample size limited the number of variables in the multiple linear regression analysis that could be adjusted for, in order to avoid overfitting. Moreover, we purposively limited the number of adjusting variables in the regression analyzes, in order to avoid adjusting for factors that potentially lie in the causal pathway between regional adiposity and cardiac remodeling. Finally, participants with previous or present cardiovascular disease were not excluded. As associations in model 2 were slightly attenuated when adjusting for self-reported cardiac disease, the observed associations between ectopic fat and LV remodeling could possibly be linked indirectly through various cardiac disorders.

## Conclusions

In the present study, the application of advanced MR-techniques permitted comprehensive characterizations of whole-body fat depots including liver fat, epicardial fat, visceral fat, abdominal subcutaneous fat, and skeletal muscle fat in participants with T2D and matched controls. Our findings show that epicardial fat is associated with LV diastolic dysfunction. The findings also propose that previously reported relations between visceral fat and LV structure could be interlinked by the levels of liver fat infiltration. On the other hand, visceral fat was associated with T2D independently of liver and abdominal subcutaneous fat. Larger follow-up studies are needed to establish these results.

## Data availability statement

The raw data supporting the conclusions of this article can be made available by the authors, upon reasonable request. Requests to access the datasets should be directed to C-JC, carl-johan.carlhall@liu.se.

## Ethics statement

The studies involving human participants were reviewed and approved by Linköping Ethical Review Board. The patients/participants provided their written informed consent to participate in this study.

## Author contributions

C-JC, JE, OL, PL, and TE conceived and planned the study. C-JC, CÖ, ES, JE, MK, OL, PL, and TE were involved in data collection. CE, MK, and TS analyzed the data. CE and C-JC carried out the statistics. CE, C-JC, ME, and OL interpreted the results. CE drafted the manuscript. All authors read and critically revised the manuscript and approved the final version.

## Funding

This work was funded by the Swedish Research Council, the Swedish Heart and Lung Foundation, and through ALF Grants Region Östergötland.

## Conflict of interest

Authors MK and OL are employees of AMRA Medical AB. Authors OL and PL are stockholders in AMRA Medical AB. Author ME reports personal fees from Advisory Board AMRA Medical AB. The remaining authors declare that the research was conducted in the absence of any commercial or financial relationships that could be construed as a potential conflict of interest.

## Publisher's note

All claims expressed in this article are solely those of the authors and do not necessarily represent those of their affiliated organizations, or those of the publisher, the editors and the reviewers. Any product that may be evaluated in this article, or claim that may be made by its manufacturer, is not guaranteed or endorsed by the publisher.

## Abbreviations

T2D: type 2 diabetes mellitus
VAT: visceral adipose tissue
ASAT: abdominal subcutaneous adipose tissue
EAT: epicardial adipose tissue
MFI: thigh muscle fat infiltration
FFMV: fat tissue-free muscle volume
BMI: body mass index
LV: left ventricular
LVEDV: left ventricular end-diastolic volume
LVM: left ventricular mass
LVC: left ventricular concentricity
EF: ejection fraction
E: early diastolic velocity of mitral inflow
A: late diastolic velocity of mitral inflow
e': early diastolic mitral annular velocity
NAFLD: non-alcoholic fatty liver disease
MRI: magnetic resonance imaging
PDFF: proton density fat fraction
PRESS: Point-RESolved Spectroscopy
SCAPIS: Swedish CArdioPulomonary bioImage Study.
