# Supplementary material for: Ectopic fat is associated with cardiac remodeling—A comprehensive assessment of regional fat depots in type 2 diabetes using multi-parametric MRI
Source: Front Cardiovasc Med. 2022 Jul 28;9:813427. doi: 10.3389/fcvm.2022.813427 (PMC9366177; doi:10.3389/fcvm.2022.813427)
Supplement: Supplementary file 1 [file Data_Sheet_1.docx]

Supplementary Material

# Data acquisition

## MRI scanning protocol

- Whole-body mDixon sequence: A total of five slabs were collected. For these, the common image parameters were TR=11 ms, TE=2.3/4.6/6.9/9.2 ms and flip angle=10°. Specific settings for slab 1-2 and 3-5 were
  - Slab 1-2 (torso): 44 slices during breath-hold, spatial resolution=1.8x1.8x5 mm^3^ and 212x151 matrix.
  - Slab 3-5 (legs): 71 slices with no breath-hold, spatial resolution=2.4x2.4x4 mm^3^, 212x213 matrix.
- Liver proton magnetic resonance spectroscopy (^1^H-MRS) PRESS sequence: TR=1500 ms, TE=35 ms, volume of interest [VOI]= c. 30x30x30 mm^3^, NSA=8.
- Cardiac 3D cine balanced steady state-free precession (bSSFP) sequence: TE=1.4 ms, TR=2.7-2.8 ms, flip angle=50°, SENSE factor 2 x 2, spatial resolution 2.5x2.5x8.0 mm^3^, acquired temporal resolution 49-69 ms, breath hold duration 16 sec, reconstructed spatial resolution 1.0x1.0x8.0 mm^3^, reconstructed heart phases 30.
- Cardiac 3D Dixon sequence: TE_1_=2.4 ms, TE_2_=4.7 ms, TR=5.7 ms, flip angle=10°, SENSE factor=2, FOV=300x300x120 mm^3^, spatial resolution=1.5x1.5x3 mm^3^, Bandwidth=541Hz/pixel, TFE factor=20, ECG-triggering to diastolic rest period, respiratory gating (5mm window), T2prep TE=40 ms.

## Echocardiography

B-mode cine loops were acquired from an apical imaging position at a frame-rate >40 fps together with pulsed-wave blood Doppler as well as tissue Doppler of wall velocity. All echocardiographic data was analyzed off-line using GE EPPC SWO v201 (GE healthcare, Horten, Norway). Diastolic functional parameters included in this sub-study were the ratio of left ventricular early diastolic filling (E) to late diastolic filling (A) and early mitral annular velocity (e´), respectively.

# Supplementary tables

## Supplementary table 1: Availability of data

Table S1 displays availability of datasets for the MRI- and echocardiography derived parameters. LV, left ventricular; E, early diastolic filling velocity; A. late diastolic filling velocity; e', early mitral annular velocity; MRS, magnetic resonance spectroscopy; PDFF, proton density fat fraction; ASAT, abdominal subcutaneous adipose tissue; FFMV, fat tissue-free thigh muscle volume; MFI, thigh muscle fat infiltration.

|  | Total (N) | T2D (N) | Controls (N) |
| --- | --- | --- | --- |
| Total participants | 92 | 46 | 46 |
| Ejection fraction | 92 | 46 | 46 |
| LV mass | 92 | 46 | 46 |
| LV end-diastolic volume | 92 | 46 | 46 |
| LV concentricity | 92 | 46 | 46 |
| E/A-ratio | 92 | 46 | 46 |
| E/e'-ratio | 79 | 38 | 41 |
| EAT | 92 | 46 | 46 |
| Liver PDFF (MRS) | 92 | 46 | 46 |
| Visceral adipose tissue | 86 | 43 | 43 |
| ASAT | 85 | 42 | 43 |
| FFMV | 84 | 42 | 42 |
| MFI | 69 | 37 | 32 |
